# Blood pressure reduction and anti‐hypertensive treatment choice: A post‐hoc analysis of the SPRINT trial

**DOI:** 10.1002/clc.23591

**Published:** 2021-04-06

**Authors:** João Pedro Ferreira, John Gregson, Michael Böhm, Patrick Rossignol, Faiez Zannad, Stuart J. Pocock

**Affiliations:** ^1^ Université de Lorraine, Centre d'Investigations Cliniques Plurithématique Inserm 1433, CHRU de Nancy Inserm U1116, FCRIN INI‐CRCT Nancy France; ^2^ Department of Medical Statistics London School of Hygiene and Tropical Medicine London UK; ^3^ Klinik für Innere Medizin III Saarland University Medical Center Homburg Germany

**Keywords:** hypertension, outcomes, treatment choices

## Abstract

**Background:**

Uncontrolled blood pressure (BP) increases the risk of major adverse cardiovascular events. In SPRINT an intensive versus standard BP lowering strategy resulted in a lower rate of cardiovascular events and death. Whether BP reduction only or also the choice of anti‐hypertensive drugs is associated with outcomes remains to be elucidated.

**Aims:**

We aim to study the association of BP and different anti‐hypertensive drugs with several cardiovascular outcomes.

**Methods:**

Time‐updated Cox and mixed‐effects models. The primary outcome was a composite of first myocardial infarction, acute coronary syndrome, stroke, heart failure, or cardiovascular death.

**Results:**

A total of 9361 patients were included. The anti‐hypertensive agents most frequently used were ACEi/ARBs, with an almost 20% higher prescription rate in the intensive arm (80% vs. 61%), followed by thiazide‐type diuretics (65% vs. 42%), calcium‐channel blockers (57% vs. 39%), and beta‐blockers (52% vs. 26%). Mineralocorticoid receptor antagonists were rarely used (≤7% of the observations). In multivariate analysis, the use of ACEi/ARBs, especially in combination with thiazides, were independently associated with a lower primary outcome event‐rate (HR [95%CI] 0.75 [0.61–0.92], *p* = .006), whereas a DBP <60 mmHg was independently associated with a higher event‐rate (HR [95%CI] 1.36 [1.07–1.71], *p* = .011). SBP <120 mmHg was associated with lower rate of cardiovascular and all‐cause death on intensive treatment but not on the standard arm (interaction *p* < .05 for both).

**Conclusions:**

In SPRINT, an intensive therapy strategy achieving SBP <120 mmHg with a DBP ≥60 mmHg, and using ACEi/ARBs plus thiazides was associated with a lower event‐rate.

## 1 INTRODUCTION

Hypertension (HT) is the most prevalent cardiovascular disease worldwide.^1^ If left uncontrolled, HT increases the risk of major adverse cardiovascular events (MACE), such as myocardial infarction (MI), stroke, heart failure (HF), chronic kidney disease (CKD), end stage renal disease, and ultimately cardiovascular (CV) death.^2^

Prior to the results of SPRINT (randomized trial of intensive versus standard blood‐pressure control),^3^ a target systolic blood pressure (SBP) of 140 mmHg or less would be generally recommended, regardless of the presence of diabetes.^4^, ^5^, ^6^ The SPRINT trial compared the benefit of treatment of SBP to a target of less than 120 mmHg (intensive) with treatment to a target of less than 140 mmHg (standard) in 9361 people aged 50 or more years, with an increased cardiovascular risk, but without diabetes or prior stroke. At 1 year, the mean SBP was 121 mmHg in the intensive group and 136 mmHg in the standard group. After a median follow‐up of 3.3 years, the trial was stopped due to a significantly lower event rate of the primary composite outcome of MI, stroke, incident HF or CV death with intensive treatment in the time‐to‐first event analysis. This effect was mainly driven by a reduction in incident HF and CV death. Moreover, an intensive treatment strategy was deemed beneficial and with a favorable benefit–risk profile in most patients included in SPRINT.^7^ However, it is not clear whether this observed effect was driven by the lower blood pressure (BP) or by other factors, such as the more frequent use of certain medications (e.g., inhibitors of the renin‐angiotensin‐aldosterone system [RAASi]) in the intensive group that may delay HF onset.^8^

In this analysis we aim to study the association of BP and anti‐HT medications with the outcomes of the SPRINT trial. We hypothesize that both a targeted BP control and the more frequent use of certain medications might have contributed to the observed risk reduction in the intensive treatment arm.

## 2 METHODS

### 2.1 Trial oversight

SPRINT was sponsored by the NHLBI, with co‐sponsorship by the National Institute of Diabetes and Digestive and Kidney Diseases, the National Institute of Neurological Disorders and Stroke, and the National Institute on Aging. The rationale and protocol for the trial have been previously published and are publicly available.^3^, ^9^ Ethics approval was obtained from each centre participating the trial. All patients signed an informed consent form to participate in the trial. SPRINT is registered in ClinicalTrials.gov with the identifier: NCT01206062.

To participate in SPRINT, patients were required to meet all the following criteria: an age of at least 50 years, a SBP of 130–180 mm Hg, and an increased risk of cardiovascular events (defined by one or more of the following: clinical or subclinical cardiovascular disease other than stroke; CKD with an estimated glomerular filtration rate [eGFR] of 20–60 ml/min/1.73 m^2^ calculated with the use of the four‐variable modification of diet in renal disease equation^10^; a 10‐year risk of cardiovascular disease ≥15% on the basis of the Framingham risk score; or an age ≥75 years). Patients with diabetes mellitus or prior stroke were excluded. All participants provided written informed consent. Participants and study personnel were aware of the study‐group assignments, but outcome adjudicators were not. The protocol encouraged, but did not mandate, the use of drug classes with the strongest evidence for reduction in cardiovascular outcomes, including thiazide‐type diuretics (encouraged as the first‐line agent), loop diuretics (for participants with advanced CKD), and beta‐adrenergic blockers (for those with coronary artery disease). Chlorthalidone was encouraged as the primary thiazide‐type diuretic, and amlodipine as the preferred calcium channel blocker (CCB). Azilsartan and azilsartan combined with chlorthalidone were donated by Takeda Pharmaceuticals International and Arbor Pharmaceuticals; neither company had any other role in the study.^11^ BP was considered as the mean of three BP measurements at an office visit while the patient was seated after 5 minutes of quiet rest; the measurements were made with the use of an automated measurement system (Model 907, Omron Healthcare). The preferred method was the automated device as it offers reduced potential for observer biases and decreased demand on staff in terms of training and effort in data collection. Medical records and electrocardiograms were obtained for documentation of events. Whenever clinical site staff became aware of a death, a standard protocol was used to obtain information on the event. A total of 9361 participants were randomized in the SPRINT trial. The trial was stopped earlier than expected after analyses of the primary outcome exceeded the monitoring boundaries at two consecutive time‐points. The median follow‐up was 3.26 years.

### 2.2 Study outcomes

The primary outcome of the SPRINT trial was a composite of MI, other acute coronary syndromes (ACS), stroke, AHF, or CV death. Compared with a standard systolic BP target of <140 mmHg and intensive strategy with a <120 mmHg, reduced the primary outcome (6.8% vs. 5.2%; 1.65% per year vs. 2.19% per year; hazard ratio [HR] with intensive treatment, 0.75; 95% confidence interval [CI], 0.64–0.89; *p* < .001). The primary outcome effect was mostly driven by the effect on AHF (HR, 0.62, 95%CI, 0.45–0.84) and CV death (HR, 0.57, 95%CI, 0.38–0.85), without a statistically significant effect on MI/ACS or stroke.^3^ For the present analysis we have studied the primary outcome, its individual components and all‐cause death.

### 2.3 Statistical analysis

In descriptive analyses, continuous variables are expressed as mean ± standard deviation (SD) or median (percentile_25‐75_) based on their histogram distribution. Categorical variables are expressed as frequencies and proportions (%). Population description and comparison of patients with “events” versus “no events” was performed using independent samples *t*‐test for normally distributed continuous variables, Mann–Whitney test for skewed variables, and *χ* ^2^ test for categorical variables. To select the baseline variables with the strongest outcome association to use in the multivariable adjusted models, we performed a multivariate stepwise (forward) model with the variables with a *p* value of <.1 from the Table 1 included in the initial model with a *p* value <.05 to enter and stay the model. To attain log‐linearity, we categorized continuous variables in concordance with the prespecified categories of age, renal function, and albuminuria.^12^ We tested all variables with a *p* value of <.1 from the Table 1 for potential treatment‐by‐variable interaction with regards to the study primary outcome and none was present. The variables retained in the model were age >75 year, female sex, current smoking, eGFR <60 ml/min/1.73 m^2^, urine albumin‐to‐creatinine ratio (UACR) >30 mg/g, prior history of CV disease and the use of three or more anti‐hypertensive (anti‐HT) drugs. This baseline model had a C‐index of 0.71 and was used for adjustment in all time‐updated multivariable models. Time‐updated models were performed using the BP measurements and anti‐HT drugs throughout the follow‐up adjusted on the baseline variables above described. An interaction term between the time‐updated variable and treatment assignment (intensive vs. standard) was tested for all the studied outcomes to assess whether the prognostic associations of BP would vary by treatment allocation. Similar analysis using the average BP throughout the follow‐up (instead of the time‐updated BP) was also performed. The average BP was computed for each studied outcome taking the BP measurements available prior to the outcome of interest. We have categorized the SBP in <120, 120–140, and >140 mmHg and DBP in <60, 60–90, and >90 mmHg based on the functional form (i.e., visual inspection of “splines” and deciles) of the variable with regards to its association with the primary outcome and considering clinically relevant and readily applicable cutoffs. The individual anti‐HT drugs were retrieved from free text and grouped into clinically relevant categories: angiotensin converting enzyme inhibitors or angiotensin receptor blockers (ACEi/ARBs), thiazide‐type diuretics, CCBs, beta‐blockers, mineralocorticoid receptor antagonists (MRAs), loop diuretics, central‐acting, and vasodilating drugs. There was only a small proportion of missing data (6.4%) in the adjustment variables that we have inputted using the mean or median of the variable (as appropriate) to avoid loss of statistical power and events. All analyses were performed with the Stata® software (StataCorp. 2019. Stata Statistical Software: Release 16. College Station, TX: StataCorp LLC).

**TABLE 1:** Baseline characteristics of the SPRINT population by the occurrence or not of a primary outcome event

Characteristic	Without event	With event	*p* value
N.	8799	562	‐
Age, yr	67.7 ± 9.3	71.7 ± 10.0	<.001
Age ≥75 yr	2391 (27.2%)	245 (43.6%)	<.001
Female	3166 (36.0%)	166 (29.5%)	.002
Race			
Black	2659 (30.2%)	143 (25.4%)	<.001
Hispanic	944 (10.7%)	40 (7.1%)	
Other	167 (1.9%)	9 (1.6%)	
White	5029 (57.2%)	370 (65.8%)	
Smoking			
Never	3920 (44.6%)	202 (35.9%)	<.001
Former	3708 (42.1%)	265 (47.2%)	
Current	1148 (13.0%)	92 (16.4%)	
BMI, Kg/m^2^	29.6 ± 6.4	29.4 ± 5.9	.48
BMI >30 Kg/m^2^	3724 (42.3%)	232 (41.3%)	.63
SBP, mmHg	139.6 ± 15.5	141.2 ± 16.7	.016
SBP <120	814 (9.3%)	58 (10.3%)	.042
SBP 120–140	4129 (46.9%)	233 (41.5%)	
SBP >140	3856 (43.8%)	271 (48.2%)	
DBP, mmHg	78.3 ± 11.9	76.1 ± 12.9	<.001
DBP <60	581 (6.6%)	59 (10.5%)	.002
DBP 60–90	6943 (78.9%)	428 (76.2%)	
DBP >90	1275 (14.5%)	75 (13.3%)	
HR, bpm	65.9 ± 12.4	66.0 ± 13.3	.82
HR >75 bpm	1753 (19.9%)	126 (22.4%)	.15
eGFR, ml/min/1.73 m2	71.8 ± 20.9	65.4 ± 22.2	<.001
eGFR <60 ml/min/1.73 m2	2412 (27.4%)	234 (41.6%)	<.001
Glucose, mg/dl	98.4 ± 15.0	99.3 ± 13.5	.15
Total cholesterol, mg/dl	189.5 ± 42.7	186.7 ± 45.2	.13
HDL‐cholesterol, mg/dl	52.8 ± 14.8	50.8 ± 14.3	.002
HDL‐cholesterol <40 mg/dl	1580 (18.0%)	127 (22.6%)	.006
Triglycerides, mg/dl	125.0 ± 91.3	132.8 ± 79.6	.047
Triglycerides >150 mg/dl	2148 (24.4%)	156 (27.8%)	.074
UACR, mg/g Cr	8.7 (5.1–19.2)	16.3 (7.1–48.4)	<.001
UACR >30 mg/g Cr	1529 (17.4%)	193 (34.3%)	<.001
History of CV disease	1370 (15.6%)	192 (34.2%)	<.001
N. anti‐HT agents			
0	848 (9.6%)	34 (6.0%)	<.001
1	2626 (29.8%)	127 (22.6%)	
2	3091 (35.1%)	201 (35.8%)	
3+	2234 (25.4%)	200 (35.6%)	
Statin	3762 (42.8%)	292 (52.0%)	<.001
Aspirin	4414 (50.2%)	342 (60.9%)	<.001
Assigned to intensive BP arm	4435 (50.4%)	243 (43.2%)	<.001

Abbreviations: BMI, body mass index; DBP, diastolic blood pressure; eGFR, estimated glomerular filtration rate; F‐U, follow‐up; HDL, high density lipoprotein; HR, heart rate; SBP, systolic blood pressure; UACR, urinary albumin‐to‐creatinine ratio.

## 3 RESULTS

### 3.1 Patients' characteristics

Patients who had a primary outcome event (n = 562) were older, more often male, white, and current smokers, had higher baseline SBP and lower DBP, had lower eGFR, lower HDL‐cholesterol, and higher UACR, they used a higher number of anti‐HT agents, statins and aspirin (Table 1). The mean BP during the follow‐up was higher in patients who had a primary outcome event (Table S1).

### 3.2 Independent risk factors

The baseline risk characteristics independently associated with higher primary outcome rate were older age (>75 year), current smoker, CKD (eGFR<60 ml/min/1.73 m^2^), UACR (>30 mg/g), history of CV disease and use of three or more anti‐HT agents. Female sex was independently associated with a lower event rate (Table S2).

### 3.3 Follow‐up time variables

#### 3.3.1 Use of anti‐hypertensive treatments

In a total of 129 565 observations (64 376 in the standard vs. 65 189 in the intensive arm), the anti‐HT drugs most frequently used were ACEi/ARBs, with an almost 20% higher prescription rate in the intensive arm (61% vs. 80%), followed by thiazide‐type diuretics, also with a near to 25% higher prescription rate in the intensive arm (42% vs. 65%). In consequence, the combination of ACEi/ARBs plus thiazides was used twice more frequently in the intensive arm (26% vs. 52%). Beta‐blockers and CCBs (alone or in combination with ACEi/ARBs) were also used more frequently in the intensive arm (33% vs. 43% and 39% vs. 57%, respectively). Centrally‐acting drugs and vasodilators were less often used, but more frequently in the intensive arm (7% vs. 14%). MRAs were used in low proportion, but with higher proportion in the intensive arm (4% vs. 7%). Loop diuretics were used at nearly similar proportions in both arms (6% vs. 7%). The use of three or more anti‐HT medications was much more frequent in the intensive arm than in the standard one (Table 2).

**TABLE 2:** Use of anti‐hypertensive drugs throughout the follow‐up

Drug	Person‐time				*p* value
	Standard	%	Intensive	%	
ACEi/ARBs	39 466	61.3	52 034	79.8	<.001
Thiazide diuretics	26 945	41.9	42 575	65.3	<.001
CCBs	25 099	39	37 399	57.4	<.001
Beta‐blockers	21 466	33.3	27 852	42.7	<.001
Central/vasodilators	4659	7.2	9164	14.1	<.001
Loop diuretics	3603	5.6	4822	7.4	<.001
MRAs	2338	3.6	4701	7.2	<.001
ACEi/ARBs plus Thiazide	16 873	26.2	34 119	52.3	<.001
ACEi/ARBs plus CCBs	15 688	23.7	29 711	44.6	<.001
ACEi/ARBs plus Beta‐blockers	13 452	20.3	21 733	32.6	<.001
Number of anti‐HT drugs					
0	4537	6.9	313	0.5	<.001
1	19 649	29.7	6337	9.5	
2	23 340	35.3	21 815	32.7	
3	13 915	21.0	23 276	34.9	
4+	4745	7.2	14 895	22.4	
Total	64 376		65 189		

*Note*: Number and proportion of time points at which a patient is on a given treatment.

Abbreviations: ACEi/ARBs, angiotensin converting enzyme inhibitors; anti‐HT, anti‐hypertensive; CCBs, calcium‐channel blockers; MRA, mineralocorticoid receptors antagonist.

### 3.4 Outcome associations

In univariate analysis, a SBP <120 mmHg (both time‐updated and average), the use of ACEi/ARBs and thiazide‐type diuretics (or their combination) were associated with a lower primary outcome event rate, whereas DBP <60 mmHg, use of beta‐blockers and loop diuretics were associated with a higher primary outcome event rate (Table S3). In multivariate analysis, the use of ACEi/ARBs and their combination with thiazides remained independently associated with a lower primary outcome event rate, whereas a DBP <60 mmHg, the use of beta‐blockers and loop diuretics remained independently associated with a higher event rate (Table 3). The association of high BP with outcomes is stronger with the average BP values than with the time‐updated BP values, because the former represents patients that had, on average, high BP during the follow‐up, whereas the time‐updated models only take into account last measure before the event.

**TABLE 3:** Association of blood pressure with the composite of myocardial infarction, stroke, heart failure hospitalization, or cardiovascular death

Variables	Person‐time spent in each BP category, n. (%)		Adjusted HR (95%CI)	*p* value	Interaction *p* ^a^
	Standard	Intensive			
Time‐updated SBP (mmHg)	‐	‐	‐	‐	.27
<120	7954 (12.4)	35 481 (54.4)	0.84 (0.67–1.05)	.12	
120–140	37 414 (58.1)	22 345 (34.3)	Referent	‐	
>140	19 008 (29.5)	7363 (11.3)	0.98 (0.78–1.22)	.84	
Time‐updated DBP (mmHg)	‐	‐	‐	‐	.99
<60	6260 (9.7)	14 449 (22.2)	1.36 (1.07–1.71)	.011	
60–90	52 809 (82.0)	48 828 (74.9)	Referent	‐	
>90	5307 (8.2)	1912 (2.9)	1.01 (0.67–1.52)	.96	
Average follow‐up SBP (mmHg)^b^	‐	‐	‐	‐	.001
<120	1273 (2.0)	26 138 (40.1)	0.77 (0.58–1.01)	.062	
120–140	50 470 (78.4)	37 096 (56.9)	Referent	‐	
>140	12 633 (19.6)	1955 (3.0)	1.46 (1.17–1.83)	.001	
Average follow‐up DBP (mmHg)^b^	‐	‐	‐	‐	.77
<60	3267 (5.1)	8958 (13.7)	1.16 (0.87–1.54)	.32	
60–90	59 011 (91.7)	55 995 (85.9)	Referent	‐	
>90	2098 (3.3)	236 (0.4)	2.18 (1.38–3.44)	.001	
Time‐updated anti‐HT drugs^c^	‐	‐	‐	‐	‐
ACEi/ARBs	39 466 (61.3)	52 034 (79.8)	0.81 (0.67–0.98)	.026	.63
Beta‐blocker	21 466 (33.3)	27 852 (42.7)	1.27 (1.05–1.53)	.014	.40
Thiazide diuretic	26 945 (41.9)	42 575 (65.3)	0.85 (0.71–1.03)	.094	.15
Calcium‐channel blocker	25 099 (39.0)	37 399 (57.4)	1.04 (0.87–1.24)	.68	.78
Loop diuretic	3603 (5.6)	4822 (7.4)	1.75 (1.36–2.26)	<.001	.91
ACEi/ARBs/Thiazide combination^b^	16 873 (26.2)	34 119 (52.3)	0.75 (0.61–0.92)	.006	.58
ACEi/ARBs/CCB combination^b^	15 688 (23.7)	29 711 (44.6)	0.95 (0.78–1.15)	.61	.41
ACEi/ARBs/BB combination^b^	13 452 (20.3)	21 733 (32.6)	0.98 (0.81–1.20)	.87	.40

*Note*: Model adjusted on the baseline variables: age >75 year, female sex, current smoking, eGFR <60 ml/min/1.73 m^2^, urine albumin‐to‐creatinine ratio >30 mg/g, prior history of CV disease, the use of 3 or more anti‐hypertensive drugs, blood pressure (systolic and diastolic) plus a treatment‐by‐variable of interest interaction term.

Abbreviations: ACEi/ARBs, angiotensin converting enzyme inhibitors; anti‐HT, anti‐hypertensive; BB, beta‐blockers; BP, blood pressure; CCB, calcium‐channel blockers; DBP, diastolic blood pressure; SBP, systolic blood pressure.

^a^ Interaction term between the time‐updated variable and treatment allocation (intensive vs. control). A statistically significant interaction means that an average systolic blood pressure <120 mmHg was associated with a lower event rate in patients on intensive therapy but not in patients on standard therapy where the event rate was increased with an average systolic blood pressure <120 mmHg. See also the Figure 1 and the Table S5.

^b^ The combination of ACE/ARBs plus Thiazides, CCB and BB was assessed in separate models that is, without the individual ACEi/ARBs, thiazides, CCB, or BB, as appropriate for each case. The average blood pressure was assessed in separate models not including the time‐updated blood pressure measurements.

^c^ The person‐time spent taking each drug is also presented in the Table 2.

**FIGURE 1:**
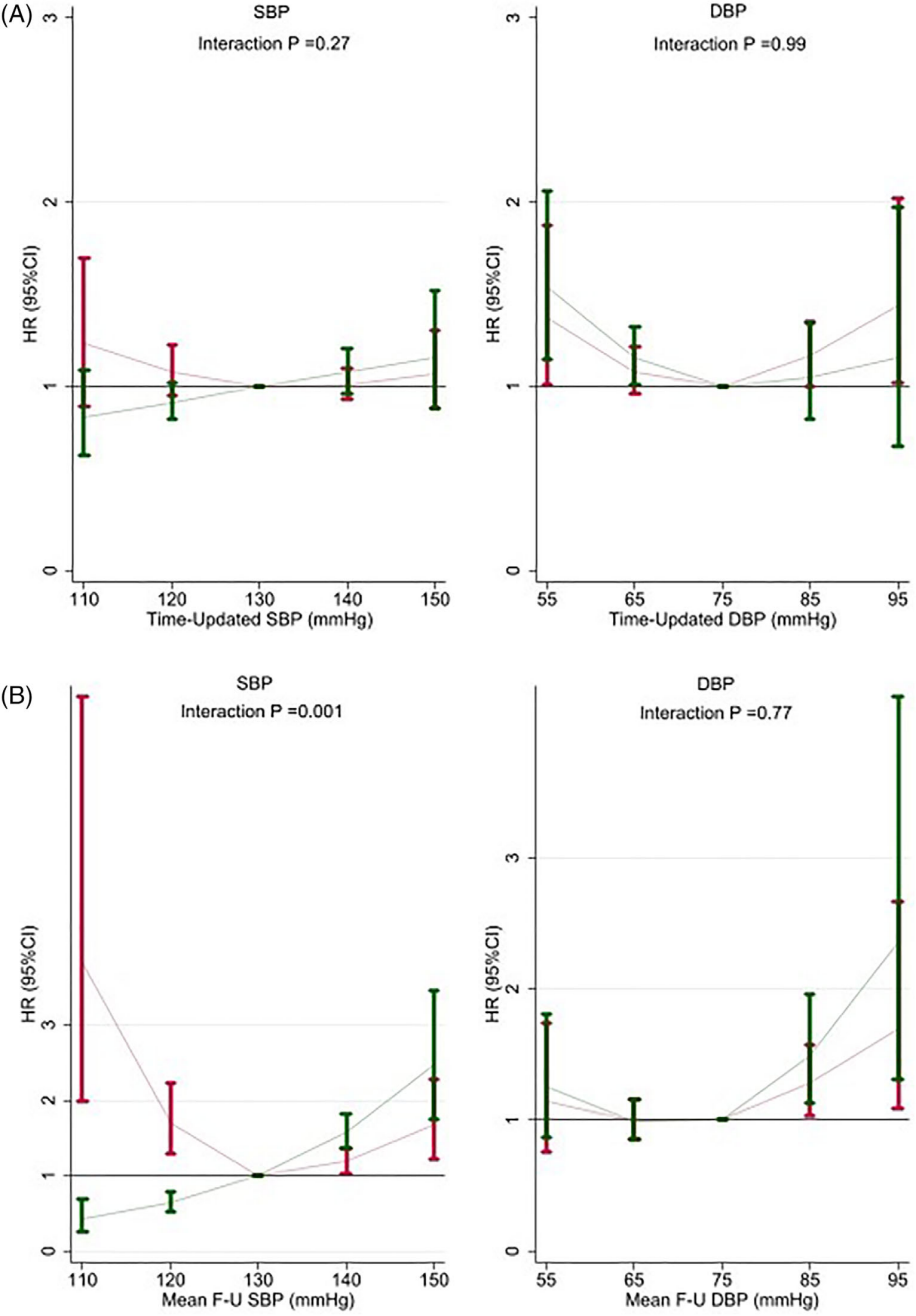
Blood pressure association with the composite of myocardial infarction, stroke, heart failure hospitalization or cardiovascular death. (A) Time‐updated (achieved) blood pressure. (B) Average blood pressure. Red bars, standard treatment; Green bars, intensive treatment. The association of SBP with the primary outcome was different between treatment groups (intensive vs. control). In the intensive group a lower achieved SBP was associated with a lower event rate; this association was particularly marked using the average SBP. Despite not reaching a statistically significant interaction in the time‐updated SBP primary outcome model, the association was directionally similar to the average SBP and significant interactions could be found for components of the primary outcome, including cardiovascular death (see also the Figure 2). DBP, diastolic blood pressure; SBP, systolic blood pressure

The association of lower SBP (<120 mmHg) with the primary outcome varied according to the treatment group allocation, particularly the average SBP (*p* for interaction =.001). A lower SBP was associated with a reduction in primary outcome events in the intensive but not in the standard arm. This differential impact on outcomes was even more marked for other end‐points (as below described under “interaction analyses”). A lower DBP (<60 mmHg) was associated with a higher event rate regardless of the treatment arm (Table 3 and Figure 1).

For the composite of CV death or HF, the use of loop diuretics remained independently associated with a higher event rate and the combination of ACEi/ARBs plus thiazides showed a tendency to be associated with a lower event rate (*p* = .076). For HF alone, a DBP <60 mmHg and the use of loop diuretics (in particular) were independently associated with a higher event rate, whereas the use of thiazides and the combination of ACEi/ARBs plus thiazides was independently associated with a lower event rate. For MI, a DBP <60 mmHg was independently associated with higher event rate, whereas the combination of ACEi/ARBs plus thiazides showed a tendency to be associated with a lower event rate (*p* = .055). For stroke a SBP >140 mmHg and a DBP <60 mmHg tended to be associated with a higher event rate (*p* = .089 and .081, respectively). Loop diuretic use was not associated with an excess of MI nor stroke. For CV death alone, only the use of loop diuretics remained associated with a higher event rate. For all‐cause death, the use of loop diuretics and beta‐blockers remained independently associated with a higher rate; whereas the use of thiazides, ACEi/ARBs or their combination were independently associated with a lower rate (Table S4).

### 3.5 Interaction analyses

**FIGURE 2:**
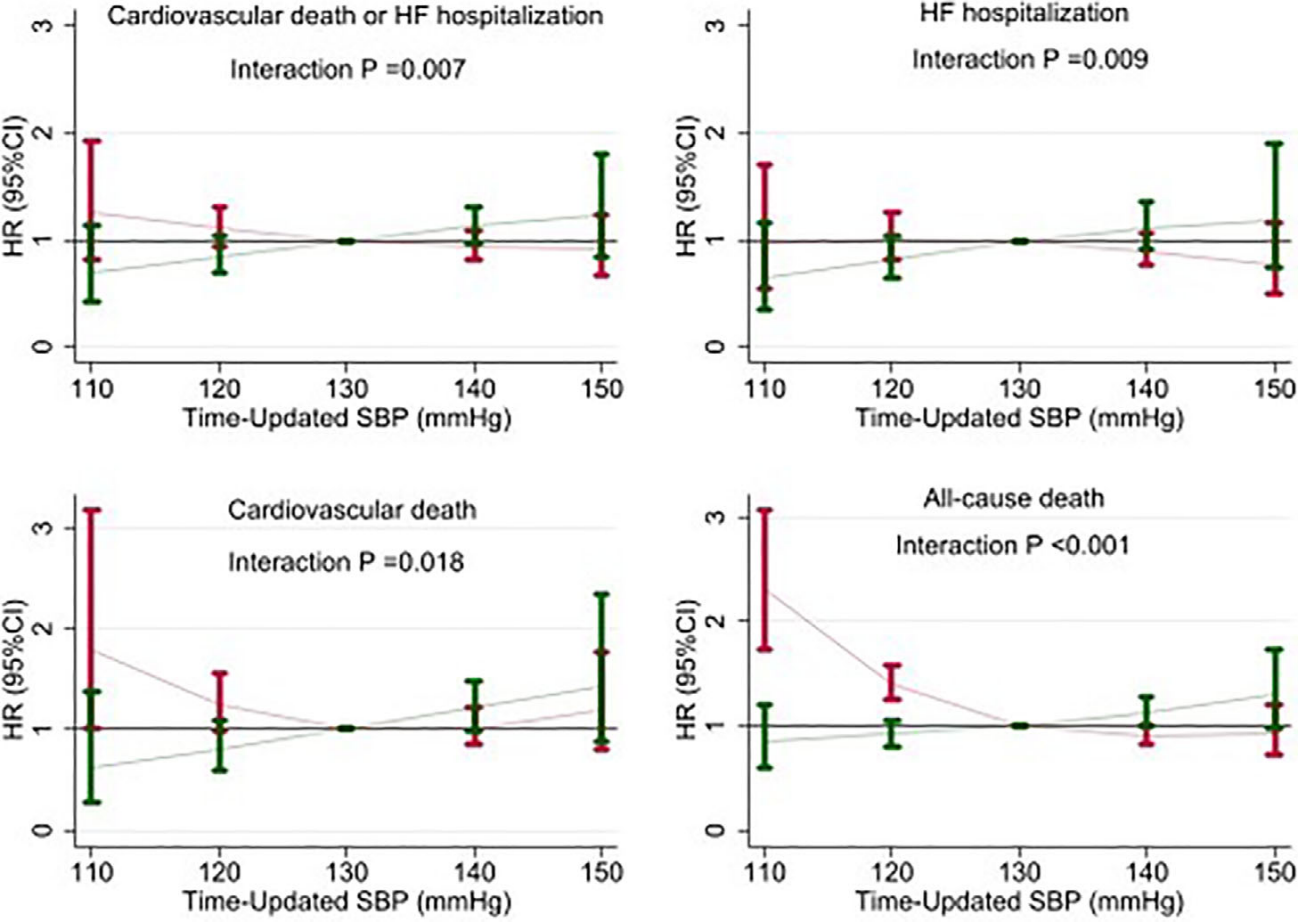
Time‐updated (achieved) systolic blood pressure by treatment interaction and its association with outcomes. Red bars, standard treatment; Green bars, intensive treatment. The association of SBP with cardiovascular death, heart failure and all‐cause death was different between treatment groups (intensive vs. control). In the intensive group a lower SBP was associated with a lower event rate, whereas in the control group it was associated with a higher event rate; the *p* for interaction was statistically significant for all the studied outcomes. HF, heart failure; SBP, systolic blood pressure

The average and achieved SBP had a marked differential impact whether patients were on intensive treatment or standard. A lower SBP (<120 mmHg) was associated with a lower event rate on the intensive therapy arm but not on the standard therapy arm where it was associated with a higher event rate. For the outcomes of CV death or HF, CV death, and all‐cause death, a statistically significant interaction of SBP‐by‐treatment allocation (intensive vs. standard) was observed (Figure 2 and Table S5).

## 4 DISCUSSION

Our study shows that patients randomized to intensive anti‐HT treatment received ACEi/ARBs, thiazides, CCBs, and beta‐blockers more frequently; they have also received more MRAs and central‐acting/vasodilating drugs but in low absolute proportions. Importantly, the combination of ACEi/ARBs plus thiazides was used twice as frequently in patients randomized to the intensive arm than in patients randomized to standard therapy (52% vs. 26%). The combination of ACEi/ARBs plus thiazides was independently associated with a lower primary outcome event rate (and also all‐cause mortality and incident HF). Moreover, a lower SBP was associated with a lower event rates in the intensive therapy arm but not in the standard therapy where higher event rates were observed. This is a pattern often found in observational studies, likely reflecting a poorer condition of the patient rather than the BP effect itself, and strongly suggests a reverse causation effect.^13^, ^14^ On the other hand, in the intensive arm, BP was intentionally reduced by a more intensive use of anti‐HT drugs, thus the lower BP reflects more the treatment effect and less the patients' underlying condition. Notwithstanding, a lower time‐updated DBP (below 60 mmHg) was associated with poorer outcomes regardless of the treatment arm, suggesting that clinicians should be wary when a patient presents with a DBP below 60 mmHg, likely due to a potential compromise of coronary perfusion,^15^, ^16^ and argues against inverse causation as the sole explanation for the association between low DBP and outcomes. Another study using the SPRINT trial data, showed that baseline DBP was associated with the study primary outcome in a U‐shaped manner, but did not modify the effect of intensive therapy.^17^ It should be noted that a high average systolic and diastolic BP (particularly diastolic) was strongly associated with adverse outcomes, which may reflect an increased arterial stiffness among patients with high average BP.^18^

The use of loop diuretics was infrequent and not much different between the treatment arms (+1.8% in intensive); their association with adverse outcomes (incident HF in particular) suggests that these drugs were used essentially for congestion relief in the setting of HF (instead of BP control) which supports an indication bias. Similar observation could be found for beta‐blockers, that were used more often in the intensive arm (+9.4%) and were associated with a higher risk of primary outcome events (but not CV death) suggesting that these drugs might have been used for treating coronary artery disease, angina, and controlling heart rate in the context of arrhythmia, thus driving the primary outcome associations. Supporting this claim, the association of beta‐blocker use with MI/ACS remained similar as for the primary outcome (adjusted HR, 1.27, 95%CI, 0.97–1.65, *p* = .081) despite the inherent loss of statistical power for the study of this outcome alone, suggesting that beta‐blockers were used more often in patients with coronary artery disease and angina pectoris (as also encouraged in the trial protocol). Thus, similar to loop diuretic use, an indication bias is likely present with beta‐blockers, and the association between beta‐blocker use and worse outcomes may be driven by their use in more severe patients.

The use of MRAs was infrequent in both groups (3.6% vs. 7.2%) and had no impact on outcomes. Given the results of PATHWAY‐2 (Spironolactone vs. placebo, bisoprolol, and doxazosin to determine the optimal treatment for drug‐resistant HT: a randomized, double‐blind, crossover trial),^19^ the role of MRAs for both controlling BP and improve CV outcomes in patients with high cardiovascular risk but without resistant HT deserves further study.^20^

The use of a fixed combination of an ARB (candesartan) plus a thiazide (hydrochlorothiazide) has been tested in the HOPE‐3 (Blood‐Pressure Lowering in Intermediate‐Risk Persons without Cardiovascular Disease) trial,^21^ where the drug association reduced BP by −6/−3 mmHg, but did not reduce MACE. However, in a prespecified subgroup of patients with high SBP (>143.5 mmHg) the treatment resulted in lower rates of MACE (of both the first and second coprimary outcomes).^21^ It should be noted that the HOPE3 population had a lower baseline risk and BP than the SPRINT population, and, in consequence, the event rate was lower, particularly for HF (×4 higher in SPRINT: 0.5% in the placebo arm of HOPE3 vs. 2.1% in the standard arm of SPRINT). These differences might help explaining the lack of effect of candesartan plus hydrochlorothiazide in the overall population, but with a beneficial effect in patients with higher baseline SBP. Another trial that can help in the dissection of our findings is the antihypertensive and lipid‐lowering treatment to prevent heart attack trial (ALLHAT),^22^ which results also demonstrated the importance of the antihypertensive class used. In ALLHAT participants were randomly assigned to receive chlorthalidone, amlodipine, or lisinopril, and showed that thiazide‐type diuretics might have delayed HF onset.^22^ Beyond BP lowering the potential advantage of associating an ACEi/ARBs with a thiazide‐type diuretic is to avoid diuretic‐induced hypokalemia, which may be harmful and potentially life‐threatening.^23^ These findings are in concordance with previous trials, where the use of ACEi reduced cardiovascular events (including HF) in patients with a high cardiovascular risk but without systolic impairment or HF.^24^, ^25^ One should also note that the greater use of ACEi/ARBs plus thiazide diuretics in the intensive treatment arm of the SPRINT study might have resulted in more pronounced alterations in intrarenal hemodynamics, leading to a rise in serum creatinine. However, this is a functional and reversible phenomenon that is seldom associated with dismal outcomes and did not decrease the benefit of intensive treatment in SPRINT.^7^, ^26^, ^27^ Furthermore, in the STOP‐HF (natriuretic peptide‐based screening and collaborative care for HF) trial, participants in the intervention group received RAASi more often reducing the combined rates of left ventricular systolic dysfunction, diastolic dysfunction, and HF.^8^ Notwithstanding, the valsartan antihypertensive long‐term use evaluation (VALUE) trial did not support a superiority of the ARB valsartan over amlodipine for reducing morbidity and mortality in hypertensive patients at high cardiovascular risk.^28^ In SPRINT, ACEi/ARBs were used much more frequently than CCBs and often in combination with CCBs, particularly in the intensive therapy arm.

Importantly, the specific treatment choices were made by the treating physician and one cannot ascertain whether ACEi/ARBs are superior to CCBs or other agents from these non‐randomized data. However, the associations reported here are of clinical relevance, because they support the choices made by most clinicians when selecting the anti‐hypertensive treatments to give to their patients.

### 4.1 Limitations

Several limitations should be acknowledged in the present analysis. First, this is a retrospective, non‐randomized and non‐prespecified analysis of a randomized controlled trial, hence the limitations inherent to observational reports are here applied. Importantly, we report associations and causation cannot be inferred from these analyses. Second, the results reported in the present analysis can only be generalized to patients with similar profile to those included in the SPRINT trial, particularly those without diabetes or stroke. Third, we were unable to determine whether the drug dose could influence the studied associations due to a high proportion of missing doses and probable value misassignment. Fourth, the direct and indirect effects of the BP and the anti‐HT treatment cannot be “mediated” due to a direct effect of the anti‐HT treatment on BP and the differential BP impact on outcomes in the intensive versus standard arms. By the associations found, both a lower SBP (if the DBP is kept above 60 mmHg) and the use of ACEi/ARBs/thiazide combination have a favorable outcome impact. Fifth, SPRINT was an open‐label strategy trial, where patients in the intensive arm had more medical visits which might have influenced the trial results in favor of the intensive arm. Lastly, most patients that participated in SPRINT were White, and these findings may not be generalizable to other populations of hypertensive patients.

## 5 CONCLUSION

In SPRINT, an intensive therapy strategy achieving a SBP <120 mmHg with a DBP ≥60 mmHg, and using ACEi/ARBs plus thiazides was associated with a lower event rate. These findings may help guide BP goals and choosing anti‐hypertensive treatments in patients with HT without diabetes.

## CONFLICT OF INTEREST

The authors have no conflicts of interest to report regarding the content of this manuscript. The content of this manuscript is solely the responsibility of the authors and does not necessarily represent the official views of the National Institutes of Health (NIH), the Department of Veterans Affairs, or the U.S. Government.

## Supporting information


**Table S1.** Average blood pressure during the follow‐up and its changes from first to the last visit.
**Table S2.** Association of baseline variables with the primary outcomes in univariate and multivariate analyses.
**Table S3.** Time updated univariate analysis for the primary outcome.
**Table S4.** Multivariable outcome associations (for outcomes other than the primary).
**Table S5.** Adjusted association of systolic blood pressure with outcomes on standard and intensive groups, separately.
