# Supplementary material for: Blood pressure reduction and anti‐hypertensive treatment choice: A post‐hoc analysis of the SPRINT trial
Source: Clin Cardiol. 2021 Apr 6;44(5):665–74. doi: 10.1002/clc.23591 (PMC8119807; doi:10.1002/clc.23591)
Supplement: Supplementary file 1 — Table S1. Average blood pressure during the follow‐up and its changes from first to the last visit. Table S2. Association of baseline variables with the primary outcomes in univariate and multivariate analyses. Table S3. Time updated univariate analysis for the primary outcome. Table S4. Multivariable outcome associations (for outcomes other than the primary). Table S5. Adjusted association of systolic blood pressure with outcomes on standard and intensive groups, separately. [file CLC-44-665-s001.docx]

**Supplemental Material**

Supplemental Table 1. Average blood pressure during the follow-up and its changes from first to the last visit

| **Average BP during the F-U** | **BP in mmHg** | **BP in mmHg** | **P-value** |
| --- | --- | --- | --- |
| Average SBP during F-U, mmHg | 129.4 ± 9.9 | 131.2 ± 10.5 | <0.001 |
| Average DBP during F-U, mmHg | 72.4 ± 9.2 | 70.1 ± 10.1 | <0.001 |

Legend: SBP, systolic blood pressure; DBP, diastolic blood pressure; F-U, follow-up.

Supplemental Table 2. Association of baseline variables with the primary outcomes in univariate and multivariate analyses

| **Univariate variable** | **HR** | **LCI** | **UCI** | **P-value** |  |
| --- | --- | --- | --- | --- | --- |
| Intensive vs. Control | 0.75 | 0.64 | 0.89 | 0.001 |  |
| Age>75yr | 2.16 | 1.83 | 2.55 | <0.001 |  |
| Female sex | 0.75 | 0.63 | 0.9 | 0.002 |  |
| Race |  |  |  |  |  |
| White | Ref. |  |  |  |  |
| Black | 0.74 | 0.61 | 0.9 | 0.002 |  |
| Hispanic | 0.58 | 0.42 | 0.81 | 0.001 |  |
| Other | 0.78 | 0.4 | 1.51 | 0.47 |  |
| Smoking |  |  |  |  |  |
| Never | Ref. |  |  |  |  |
| Former | 1.37 | 1.14 | 1.64 | 0.001 |  |
| Current | 1.55 | 1.21 | 1.98 | 0.001 |  |
| BMI>30Kg/m2 | 0.94 | 0.8 | 1.12 | 0.51 |  |
| SBP (mmHg) |  |  |  |  |  |
| <120 | 1.26 | 0.94 | 1.67 | 0.12 |  |
| 120-140 | Ref. |  |  |  |  |
| >140 | 1.25 | 1.05 | 1.49 | 0.013 |  |
| DBP (mmHg) |  |  |  |  |  |
| <60 | 1.7 | 1.29 | 2.22 | <0.001 |  |
| 60-90 | Ref. |  |  |  |  |
| >90 | 0.96 | 0.75 | 1.23 | 0.76 |  |
| HR>75bpm | 1.17 | 0.96 | 1.43 | 0.12 |  |
| eGFR<60ml/min | 1.84 | 1.55 | 2.17 | <0.001 |  |
| HDLc<40mg/dL | 1.33 | 1.09 | 1.62 | 0.004 |  |
| TG>150mg/dL | 1.16 | 0.96 | 1.39 | 0.12 |  |
| UACr>30mg/g | 2.48 | 2.09 | 2.96 | <0.001 |  |
| CV disease hx | 2.76 | 2.32 | 3.29 | <0.001 |  |
| No. anti-HT agents |  |  |  |  |  |
| 0 | Ref. |  |  |  |  |
| 1 | 1.17 | 0.8 | 1.71 | 0.41 |  |
| 2 | 1.55 | 1.08 | 2.23 | 0.018 |  |
| 3+ | 2.12 | 1.47 | 3.05 | <0.001 |  |
| **Multivariate** |  |  |  |  | **Interaction P** |
| Intensive vs. Control | 0.75 | 0.63 | 0.88 | 0.001 |  |
| Age>75yr | 1.9 | 1.59 | 2.27 | <0.001 | 0.34 |
| Female sex | 0.8 | 0.67 | 0.97 | 0.02 | 0.43 |
| Current smk | 1.66 | 1.32 | 2.1 | <0.001 | 0.28 |
| eGFR<60ml/min | 1.32 | 1.11 | 1.58 | 0.002 | 0.27 |
| UACR>30mg/g | 1.98 | 1.65 | 2.37 | <0.001 | 0.9 |
| CV disease hx | 2.26 | 1.89 | 2.7 | <0.001 | 0.22 |
| 3+ anti-HT agents | 1.3 | 1.09 | 1.56 | 0.004 | 0.4 |

Legend: HR, hazard ratio; LCI, lower bound 95% confidence interval; UCI, upper bound 95% confidence interval; Interaction P, tested between each variable and the treatment arm.

Supplemental Table 3. Time updated univariate analysis for the primary outcome

| **Variable** | **HR** | **LCI** | **UCI** | **P-value** |
| --- | --- | --- | --- | --- |
| Time-updated SBP (mmHg) |  |  |  |  |
| <120 | 0.79 | 0.65 | 0.95 | 0.015 |
| 120-140 | Ref. |  |  |  |
| >140 | 1.09 | 0.89 | 1.35 | 0.41 |
| Time-updated DBP (mmHg) |  |  |  |  |
| <60 | 1.7 | 1.4 | 2.07 | <0.001 |
| 60-90 | Ref. |  |  |  |
| >90 | 1.08 | 0.74 | 1.56 | 0.69 |
| Average SBP (mmHg) |  |  |  |  |
| <120 | 0.68 | 0.53 | 0.87 | 0.002 |
| 120-140 | Ref. |  |  |  |
| >140 | 1.84 | 1.50 | 2.26 | <0.001 |
| Average DBP (mmHg) |  |  |  |  |
| <60 | 1.79 | 1.42 | 2.26 | <0.001 |
| 60-90 | Ref. |  |  |  |
| >90 | 2.20 | 1.46 | 3.32 | <0.001 |
| Time-updated anti-HT drugs |  |  |  |  |
| ACEi/ARBs | 0.82 | 0.69 | 0.98 | 0.028 |
| Beta-blocker | 1.91 | 1.62 | 2.25 | <0.001 |
| CCB | 1.14 | 0.97 | 1.35 | 0.11 |
| MRA | 0.97 | 0.67 | 1.39 | 0.86 |
| Thiazide diuretic | 0.67 | 0.57 | 0.79 | <0.001 |
| Loop diuretic | 2.84 | 2.28 | 3.55 | <0.001 |
| Central acting/Vasodilator | 1.16 | 0.9 | 1.49 | 0.25 |
| ACEi/ARBs/Thiazide combination | 0.65 | 0.54 | 0.77 | <0.001 |

Legend: HR, hazard ratio; LCI, lower bound 95% confidence interval; UCI, upper bound 95% confidence interval; anti-HT, anti-hypertensive.

Supplemental Table 4. Multivariable outcome associations (for outcomes other than the primary)

| **Multivariate** | **HR** | **LCI** | **UCI** | **P-value** | **Interaction P** |
| --- | --- | --- | --- | --- | --- |
| **Cardiovascular death or heart failure** | | | | | |
| Time-updated SBP (mmHg) |  |  |  |  |  |
| <120 | 0.85 | 0.6 | 1.2 | 0.36 |  |
| 120-140 | Ref. |  |  |  | 0.007 |
| >140 | 0.97 | 0.7 | 1.35 | 0.84 |  |
| Time-updated DBP (mmHg) |  |  |  |  |  |
| <60 | 1.31 | 0.95 | 1.82 | 0.1 |  |
| 60-90 | Ref. |  |  |  | 0.96 |
| >90 | 1.24 | 0.69 | 2.21 | 0.47 |  |
| Average SBP (mmHg) |  |  |  |  |  |
| <120 | 0.97 | 0.63 | 1.48 | 0.87 |  |
| 120-140 | Ref. |  |  |  | 0.012 |
| >140 | 1.43 | 1.04 | 1.98 | 0.030 |  |
| Average DBP (mmHg) |  |  |  |  |  |
| <60 | 0.93 | 0.63 | 1.37 | 0.72 |  |
| 60-90 | Ref. |  |  |  | 0.45 |
| >90 | 2.28 | 1.18 | 4.41 | 0.014 |  |
| Time-updated anti-HT drugs |  |  |  |  |  |
| ACEi/ARBs | 0.83 | 0.62 | 1.1 | 0.19 | 0.33 |
| Beta-blocker | 1.31 | 0.98 | 1.75 | 0.069 | 0.55 |
| Thiazide diuretic | 0.8 | 0.59 | 1.08 | 0.14 | 0.21 |
| Loop diuretic | 2.85 | 2.02 | 4.01 | <0.001 | 0.64 |
| ACEi/ARBs/Thiazide combination * | 0.74 | 0.53 | 1.03 | 0.076 | 0.7 |
| **Heart failure hospitalization** | | | | | |
| Time-updated SBP (mmHg) |  |  |  |  |  |
| <120 | 0.68 | 0.45 | 1.03 | 0.070 |  |
| 120-140 | Ref. |  |  |  | 0.009 |
| >140 | 0.81 | 0.54 | 1.22 | 0.314 |  |
| Time-updated DBP (mmHg) |  |  |  |  |  |
| <60 | 1.53 | 1.05 | 2.23 | 0.028 |  |
| 60-90 | Ref. |  |  |  | 0.7 |
| >90 | 1.01 | 0.46 | 2.26 | 0.972 |  |
| Average SBP (mmHg) |  |  |  |  |  |
| <120 | 0.91 | 0.54 | 1.53 | 0.72 |  |
| 120-140 | Ref. |  |  |  | 0.10 |
| >140 | 1.29 | 0.87 | 1.92 | 0.21 |  |
| Average DBP (mmHg) |  |  |  |  |  |
| <60 | 1.11 | 0.72 | 1.70 | 0.64 |  |
| 60-90 | Ref. |  |  |  | 0.30 |
| >90 | 1.79 | 0.71 | 4.50 | 0.21 |  |
| Time-updated anti-HT drugs |  |  |  |  |  |
| ACEi/ARBs | 0.82 | 0.58 | 1.15 | 0.245 | 0.51 |
| Beta-blocker | 1.41 | 0.98 | 2.01 | 0.061 | 0.93 |
| Thiazide diuretic | 0.62 | 0.43 | 0.91 | 0.014 | 0.21 |
| Loop diuretic | 2.81 | 1.88 | 4.20 | 0.000 | 0.61 |
| ACEi/ARBs/Thiazide combination * | 0.63 | 0.42 | 0.95 | 0.029 | 0.99 |
| **Myocardial infarction** | | | | | |
| Time-updated SBP (mmHg) |  |  |  |  |  |
| <120 | 0.85 | 0.60 | 1.20 | 0.351 |  |
| 120-140 | Ref. |  |  |  | 0.38 |
| >140 | 0.99 | 0.68 | 1.42 | 0.940 |  |
| Time-updated DBP (mmHg) |  |  |  |  |  |
| <60 | 1.49 | 1.05 | 2.11 | 0.026 |  |
| 60-90 | Ref. |  |  |  | 0.68 |
| >90 | 1.03 | 0.54 | 1.96 | 0.940 |  |
| Average SBP (mmHg) |  |  |  |  |  |
| <120 | 0.72 | 0.47 | 1.11 | 0.14 |  |
| 120-140 | Ref. |  |  |  | 0.89 |
| >140 | 1.50 | 1.05 | 2.15 | 0.025 |  |
| Average DBP (mmHg) |  |  |  |  |  |
| <60 | 1.46 | 0.99 | 2.17 | 0.059 |  |
| 60-90 | Ref. |  |  |  | 0.81 |
| >90 | 2.93 | 1.59 | 5.41 | 0.001 |  |
| Time-updated anti-HT drugs |  |  |  |  |  |
| ACEi/ARBs | 0.79 | 0.59 | 1.07 | 0.135 | 0.89 |
| Beta-blocker | 1.27 | 0.97 | 1.65 | 0.081 | 0.83 |
| Thiazide diuretic | 0.77 | 0.57 | 1.05 | 0.097 | 0.56 |
| Loop diuretic | 1.22 | 0.78 | 1.91 | 0.384 | 0.86 |
| ACEi/ARBs/Thiazide combination * | 0.73 | 0.53 | 1.01 | 0.055 | 0.85 |
| **Stroke** | | | | | |
| Time-updated SBP (mmHg) |  |  |  |  |  |
| <120 | 0.70 | 0.44 | 1.11 | 0.128 |  |
| 120-140 | Ref. |  |  |  | 0.79 |
| >140 | 1.45 | 0.94 | 2.23 | 0.089 |  |
| Time-updated DBP (mmHg) |  |  |  |  |  |
| <60 | 1.49 | 0.95 | 2.33 | 0.081 |  |
| 60-90 | Ref. |  |  |  | 0.72 |
| >90 | 0.89 | 0.40 | 1.99 | 0.775 |  |
| Average SBP (mmHg) |  |  |  |  |  |
| <120 | 0.64 | 0.36 | 1.14 | 0.13 |  |
| 120-140 | Ref. |  |  |  | 0.004 |
| >140 | 2.39 | 1.57 | 3.64 | <0.001 |  |
| Average DBP (mmHg) |  |  |  |  |  |
| <60 | 1.39 | 0.84 | 2.30 | 0.20 |  |
| 60-90 | Ref. |  |  |  | 0.68 |
| >90 | 1.69 | 0.67 | 4.27 | 0.27 |  |
| Time-updated anti-HT drugs |  |  |  |  |  |
| ACEi/ARBs | 0.80 | 0.55 | 1.18 | 0.263 | 0.12 |
| Beta-blocker | 1.24 | 0.85 | 1.82 | 0.267 | 0.51 |
| Thiazide diuretic | 1.04 | 0.71 | 1.51 | 0.841 | 0.21 |
| Loop diuretic | 0.68 | 0.34 | 1.36 | 0.277 | 0.4 |
| ACEi/ARBs/Thiazide combination * | 0.79 | 0.53 | 1.18 | 0.251 | 0.51 |
| **Cardiovascular death** | | | | | |
| Time-updated SBP (mmHg) |  |  |  |  |  |
| <120 | 1.07 | 0.62 | 1.83 | 0.811 |  |
| 120-140 | Ref. |  |  |  | 0.018 |
| >140 | 1.55 | 0.96 | 2.51 | 0.074 |  |
| Time-updated DBP (mmHg) |  |  |  |  |  |
| <60 | 1.22 | 0.73 | 2.04 | 0.456 |  |
| 60-90 | Ref. |  |  |  | 0.58 |
| >90 | 1.32 | 0.61 | 2.89 | 0.481 |  |
| Average SBP (mmHg) |  |  |  |  |  |
| <120 | 1.02 | 0.53 | 1.96 | 0.94 |  |
| 120-140 | Ref. |  |  |  | 0.014 |
| >140 | 2.14 | 1.35 | 3.41 | 0.001 |  |
| Average DBP (mmHg) |  |  |  |  |  |
| <60 | 1.08 | 0.61 | 1.93 | 0.79 |  |
| 60-90 | Ref. |  |  |  | 0.74 |
| >90 | 3.59 | 1.59 | 8.10 | 0.002 |  |
| Time-updated anti-HT drugs |  |  |  |  |  |
| ACEi/ARBs | 0.79 | 0.51 | 1.21 | 0.280 | 0.27 |
| Beta-blocker | 1.29 | 0.83 | 2.01 | 0.255 | 0.4 |
| Thiazide diuretic | 1.36 | 0.86 | 2.14 | 0.193 | 0.48 |
| Loop diuretic | 4.55 | 2.70 | 7.67 | 0.000 | 0.83 |
| ACEi/ARBs/Thiazide combination * | 1.05 | 0.64 | 2.03 | 0.85 | 0.79 |
| **All-cause death** | | | | | |
| Time-updated SBP (mmHg) |  |  |  |  |  |
| <120 | 1.20 | 0.92 | 1.57 | 0.172 |  |
| 120-140 | Ref. |  |  |  | <0.001 |
| >140 | 1.23 | 0.94 | 1.61 | 0.139 |  |
| Time-updated DBP (mmHg) |  |  |  |  |  |
| <60 | 1.28 | 0.98 | 1.66 | 0.067 |  |
| 60-90 | Ref. |  |  |  | 0.21 |
| >90 | 1.15 | 0.71 | 1.86 | 0.573 |  |
| Average SBP (mmHg) |  |  |  |  |  |
| <120 | 1.00 | 0.73 | 1.37 | 0.99 |  |
| 120-140 | Ref. |  |  |  | <0.001 |
| >140 | 1.42 | 1.08 | 1.86 | 0.012 |  |
| Average DBP (mmHg) |  |  |  |  |  |
| <60 | 1.15 | 0.85 | 1.55 | 0.36 |  |
| 60-90 | Ref. |  |  |  | 0.30 |
| >90 | 2.13 | 1.22 | 3.70 | 0.008 |  |
| Time-updated anti-HT drugs |  |  |  |  |  |
| ACEi/ARBs | 0.73 | 0.58 | 0.91 | 0.005 | 0.45 |
| Beta-blocker | 1.30 | 1.03 | 1.65 | 0.025 | 0.54 |
| Thiazide diuretic | 0.78 | 0.61 | 0.98 | 0.037 | 0.15 |
| Loop diuretic | 2.31 | 1.73 | 3.08 | 0.000 | 0.34 |
| ACEi/ARBs/Thiazide combination * | 0.72 | 0.55 | 0.94 | 0.015 | 0.55 |

Legend: HR, hazard ratio; LCI, lower bound 95% confidence interval; UCI, upper bound 95% confidence interval; anti-HT, anti-hypertensive.

Models adjusted on baseline treatment allocation (intensive vs. control), age (≤75yr vs. >75yr), sex, smoking, renal function (eGFR <60ml/min vs. ≥60ml/min), albuminuria (UACR≤30mg/g vs. >30mg/g), history of cardiovascular disease, use of 3 or more anti-HT agents at baseline.

For statistically significant interactions, see also the Supplemental Table 5 depicting the differential blood pressure impact on intensive and standard therapy, separately.

Supplemental Table 5. Adjusted association of systolic blood pressure with outcomes on standard and intensive groups, separately

| **SBP (mmHg)** | **Standard** | | | | **Intensive** | | | |  |
| --- | --- | --- | --- | --- | --- | --- | --- | --- | --- |
|  | **HR** | **LCI** | **UCI** | **P-value** | **HR** | **LCI** | **UCI** | **P-value** | **InteractionP** |
| **Primary outcome** |  |  |  |  |  |  |  |  |  |
| Time-updated |  |  |  |  |  |  |  |  |  |
| <120 | 1.08 | 0.77 | 1.52 | 0.64 | 0.79 | 0.59 | 1.05 | 0.10 |  |
| 120-140 | Ref. |  |  |  | Ref. |  |  |  | 0.24 |
| >140 | 0.92 | 0.71 | 1.20 | 0.54 | 1.14 | 0.76 | 1.71 | 0.52 |  |
| Average |  |  |  |  |  |  |  |  |  |
| <120 | 1.55 | 0.83 | 2.88 | 0.17 | 0.78 | 0.58 | 1.05 | 0.10 |  |
| 120-140 | Ref. |  |  |  | Ref. |  |  |  | 0.001 |
| >140 | 1.16 | 0.89 | 1.51 | 0.26 | 2.66 | 1.80 | 3.92 | <0.001 |  |
| **CV death or HF hosp.** |  |  |  |  |  |  |  |  |  |
| Time-updated |  |  |  |  |  |  |  |  |  |
| <120 | 1.35 | 0.86 | 2.12 | 0.19 | 0.62 | 0.38 | 1.02 | 0.061 |  |
| 120-140 | Ref. |  |  |  | Ref. |  |  |  | 0.007 |
| >140 | 0.85 | 0.57 | 1.26 | 0.41 | 1.37 | 0.76 | 2.47 | 0.29 |  |
| Average |  |  |  |  |  |  |  |  |  |
| <120 | 2.25 | 1.06 | 4.77 | 0.034 | 0.86 | 0.52 | 1.44 | 0.57 |  |
| 120-140 | Ref. |  |  |  | Ref. |  |  |  | 0.012 |
| >140 | 1.26 | 0.86 | 1.84 | 0.23 | 2.28 | 1.23 | 4.25 | 0.009 |  |
| **HF hospitalization** |  |  |  |  |  |  |  |  |  |
| Time-updated |  |  |  |  |  |  |  |  |  |
| <120 | 1.07 | 0.61 | 1.87 | 0.83 | 0.54 | 0.30 | 0.96 | 0.037 |  |
| 120-140 | Ref. |  |  |  | Ref. |  |  |  | 0.009 |
| >140 | 0.62 | 0.37 | 1.04 | 0.07 | 1.38 | 0.70 | 2.71 | 0.35 |  |
| Average |  |  |  |  |  |  |  |  |  |
| <120 | 1.98 | 0.77 | 5.11 | 0.16 | 0.85 | 0.46 | 1.57 | 0.60 |  |
| 120-140 | Ref. |  |  |  | Ref. |  |  |  | 0.10 |
| >140 | 1.18 | 0.74 | 1.88 | 0.49 | 1.82 | 0.85 | 3.88 | 0.12 |  |
| **Myocardial infarction** |  |  |  |  |  |  |  |  |  |
| Time-updated |  |  |  |  |  |  |  |  |  |
| <120 | 1.00 | 0.57 | 1.77 | 0.99 | 0.89 | 0.57 | 1.39 | 0.60 |  |
| 120-140 | Ref. |  |  |  | Ref. |  |  |  | 0.38 |
| >140 | 1.10 | 0.71 | 1.68 | 0.68 | 0.82 | 0.39 | 1.71 | 0.59 |  |
| Average |  |  |  |  |  |  |  |  |  |
| <120 | 0.67 | 0.16 | 2.79 | 0.59 | 0.75 | 0.47 | 1.19 | 0.22 |  |
| 120-140 | Ref. |  |  |  | Ref. |  |  |  | 0.89 |
| >140 | 1.50 | 0.99 | 2.28 | 0.058 | 1.67 | 0.82 | 3.40 | 0.15 |  |
| **Stroke** |  |  |  |  |  |  |  |  |  |
| Time-updated |  |  |  |  |  |  |  |  |  |
| <120 | 0.65 | 0.27 | 1.57 | 0.34 | 0.78 | 0.44 | 1.39 | 0.40 |  |
| 120-140 | Ref. |  |  |  | Ref. |  |  |  | 0.79 |
| >140 | 1.30 | 0.77 | 2.20 | 0.33 | 1.76 | 0.84 | 3.70 | 0.13 |  |
| Average |  |  |  |  |  |  |  |  |  |
| <120 | 0.76 | 0.10 | 5.59 | 0.79 | 0.72 | 0.39 | 1.34 | 0.30 |  |
| 120-140 | Ref. |  |  |  | Ref. |  |  |  | 0.004 |
| >140 | 1.36 | 0.80 | 2.32 | 0.26 | 6.45 | 3.34 | 12.5 | <0.001 |  |
| **Cardiovascular death** |  |  |  |  |  |  |  |  |  |
| Time-updated |  |  |  |  |  |  |  |  |  |
| <120 | 2.31 | 1.18 | 4.50 | 0.014 | 0.52 | 0.24 | 1.14 | 0.11 |  |
| 120-140 | Ref. |  |  |  | Ref. |  |  |  | 0.018 |
| >140 | 1.61 | 0.90 | 2.88 | 0.11 | 1.58 | 0.67 | 3.76 | 0.30 |  |
| Average |  |  |  |  |  |  |  |  |  |
| <120 | 2.52 | 0.86 | 7.41 | 0.093 | 0.96 | 0.43 | 2.18 | 0.93 |  |
| 120-140 | Ref. |  |  |  | Ref. |  |  |  | 0.014 |
| >140 | 1.64 | 0.94 | 2.86 | 0.080 | 5.05 | 2.18 | 11.7 | <0.001 |  |
| **All-cause death** |  |  |  |  |  |  |  |  |  |
| Time-updated |  |  |  |  |  |  |  |  |  |
| <120 | 2.54 | 1.79 | 3.60 | <0.001 | 0.65 | 0.45 | 0.93 | 0.020 |  |
| 120-140 | Ref. |  |  |  | Ref. |  |  |  | <0.001 |
| >140 | 1.24 | 0.89 | 1.74 | 0.20 | 1.35 | 0.84 | 2.16 | 0.22 |  |
| Average |  |  |  |  |  |  |  |  |  |
| <120 | 3.25 | 1.92 | 5.51 | <0.001 | 0.75 | 0.51 | 1.10 | 0.14 |  |
| 120-140 | Ref. |  |  |  | Ref. |  |  |  | <0.001 |
| >140 | 1.18 | 0.85 | 1.64 | 0.33 | 2.53 | 1.56 | 4.11 | <0.001 |  |

Legend: HR, hazard ratio; LCI, lower 95% confidence interval; UCI, upper 95% confidence interval; SBP, systolic blood pressure; HFH, heart failure hospitalization; CVD, cardiovascular death; MI, myocardial infarction; ACM, all-cause mortality; SBP, systolic blood pressure.

Models adjusted on baseline treatment allocation (intensive vs. control), age (≤75yr vs. >75yr), sex, smoking, renal function (eGFR <60ml/min vs. ≥60ml/min), albuminuria (UACR≤30mg/g vs. >30mg/g), history of cardiovascular disease, use of 3 or more anti-HT agents at baseline.
